# The impact of increasing the United Kingdom national minimum wage on self‐reported health

**DOI:** 10.1002/hec.4490

**Published:** 2022-03-31

**Authors:** Jacob Maxwell, Robert Pryce, Luke B. Wilson

**Affiliations:** ^1^ School of Health and Related Research University of Sheffield Sheffield UK

**Keywords:** minimum wage, self‐reported health

## Abstract

There is a growing but mixed literature on the health effects of minimum wages. If minimum wage changes have a statistically significant impact on health, this suggests health effects should be incorporated into cost‐benefit analyses to capture wider policy impacts. Whilst most existing UK based literature examines the introduction of a minimum wage, this paper exploits the 2016, 2017 and 2018 UK National Minimum Wage (NMW) increases as natural experiments using a series of difference‐in‐differences models. Short Form‐12 (SF‐12) mental and physical component summary scores are used as dependent variables. In the base case and all sensitivity analyses, the estimated impact of NMW increases on mental and physical health are insignificant. The policy implication is that health effects should not be included in cost‐benefit analyses examining the NMW.

## 1 INTRODUCTION

The National Minimum Wage (NMW) was introduced in the UK in April 1999 with the aim of increasing the incomes of the lowest paid in society and to address the worst cases of labor market exploitation (Low Pay Commission, 1998). Since then, the UK NMW has consistently increased in nominal terms. Given the association between disadvantage and poor health (Marmot et al., 2010), one may expect that raising minimum wages would have an impact on the health of individuals receiving wage increases.

Lenhart (2017) states that higher minimum wages may improve mental health by reducing financial stress and income insecurity. Conversely, Reeves et al. (2017) suggest that wage increases may affect the uptake of risky behaviors such as smoking or alcohol consumption. There is an existing literature which highlights the complex relationship between income and wealth and the consumption of healthy or unhealthy goods (Adda et al., 2009; van Kippersluis & Galama, 2014). In particular, “more affluent individuals are less likely to smoke, drink heavily, be overweight, and use illegal drugs, and are more likely to exercise [than less affluent groups]” (Kippersluis & Galama, 2014, p197), but they tend to engage more in some moderately unhealthy behaviors (such as moderate drinking), and less in other unhealthy behaviors (such as smoking). Kippersluis and Galama state that it is unclear what explains the differences in health behaviors across wealth groups (2014). Kenkel et al. (2014) demonstrate that cigarettes are a normal good, and therefore, an increase in income may lead to increased cigarette consumption (an unhealthy behavior). However, the same may be true of healthy behaviors such as fruit and vegetable consumption. The complex interactions between price, income, wealth and preferences are fields of research in themselves. In this paper we specifically examine the net effect on health of a change in wages (or income), and in particular, where that change in wages results in a change of purchasing power (i.e., wage changes above the rate of inflation).

There is a growing but mixed literature on the health effects of minimum wages. Reeves et al. (2017) is the first study to analyse the association between the UK NMW and mental health. The authors apply a difference‐in‐differences (DiD) approach, using the 1999 introduction of the NMW as a natural experiment, finding a positive and statistically significant impact of the NMW on mental health in their treatment group. Kronenberg et al. (2017) extend the analysis of Reeves et al. (2017) by redefining the treatment and control groups to achieve a larger sample size. However, Kronenberg et al. (2017) find an insignificant effect of the NMW on mental health. Lenhart (2017) also applies DiD to this same natural experiment, finding a positive and statistically significant relationship between the NMW introduction and self‐reported health. Reeves et al. (2017), Kronenberg et al. (2017) and Lenhart (2017) analyze the same dataset, policy intervention and all use DiD methods, but find different results. It is notable that the papers use different regression models, although the use of different models cannot fully explain the range of results found in these papers. One can conclude that the findings are sensitive to the definitions used to identify treatment groups, model type and specification, and, the dependent variable used, although decomposing these effects is challenging.

The majority of other studies are US based and typically utilise differential timings of federal and state minimum wage increases as natural experiments (Horn et al., 2017; Wehby et al., 2020). Horn et al. (2017) find that minimum wage increases lead to a higher probability of lower‐skilled individuals reporting fair or poor general health. Wehby et al. (2020) use a DiD framework and find a positive and significant causal relationship between minimum wage increases and child health. However, these causal relationships were limited to specific population subgroups. Whilst UK based studies typically analyse panel data, US studies utilise repeated cross‐sectional data and require significant testing to ensure the sample composition remains stable before and after the policy change. However, US studies have the advantage that the authors can examine a large sample of over 300 different minimum wage increases over a 23‐year period, whilst the UK papers outlined above examine only one policy change in 1999.

This paper runs a series of DiD models exploiting the 2016, 2017 and 2018 NMW increases as natural experiments, using data from The UK Household Longitudinal Study (Understanding Society). Mental and physical component summary scores from the Short Form‐12 (SF‐12), a widely used self‐reported health measure, are used as dependent variables. If the NMW has a statistically significant positive impact on health, this suggests health effects should be incorporated into cost‐benefit analyses as to capture the wider effects of the policy. The motivation stems from the fact that cost‐benefit analyses examining the uprating of the UK NMW do not examine the potential impact of the policy on health (Department for Business, Energy and Industrial Strategy, 2021; Low Pay Commission, 2020). UK government appraisal and evaluation guidance states that health effects should be included in economic evaluations if appropriate (HM Treasury, 2020). Therefore, a causal link between the NMW and health may justify its inclusion as a wider impact of the policy in subsequent NMW cost‐benefit analyses. The paper contributes to the literature in two ways. First, the paper examines the subsequent upratings of the NMW, whilst previous literature has only focused on the introduction of the NMW in 1999. The analysis therefore uses more up‐to‐date data than other UK based studies, and, allows a greater number of natural experiments to be examined (as the NMW introduction offers only one natural experiment which occurred over 20 years ago). Second, to the authors' knowledge, this is the first paper, UK based or otherwise, to examine the impact of the NMW on SF‐12 responses. This is a notable contribution in the UK context as UK studies typically use the General Health Questionnaire as a self‐reported measure of mental health, whilst the effect of the NMW on physical health is typically analysed by examining the undertaking of risky behaviors. The SF‐12 provides measures of both self‐reported mental and physical health which have not previously been analysed in similar studies.

## 2 DATA

This analysis employs data from Understanding Society, which is a nationally representative longitudinal survey of 40,000 households in the United Kingdom. Understanding Society has several advantages. Firstly, as the study is an individual‐level panel dataset, time‐invariant unobserved heterogeneity can be accounted for. Secondly, it contains detailed hourly wage information which can be used to identify individuals subject to the NMW, meaning that individuals can be accurately allocated to treatment and control groups. Similar studies have relied on derived hourly wage rates or proxy measures to allocate individuals to treatment and control groups which may be more prone to measurement errors (Horn et al., 2017; Reeves et al., 2017). Finally, all individuals aged 10 or above within the households are interviewed, meaning the survey achieves a representative sample of the income, age, and health distributions (Lenhart, 2017). A potential disadvantage is the risk of systematic reporting bias which is discussed later.

### 2.1 Identification of treatment and control groups

The sample was restricted to individuals aged 25–64 at the time of their interview. Individuals aged below 25 are excluded as from April 2016 this is the eligibility age for the top NMW rate. Those aged 65 and above are excluded as this is the age at which individuals become eligible for pension benefits, such as the state pension. This increase in income may be a confounding factor affecting health, so was removed. Individuals must be employed in both periods $t_{b}$ (before the policy change) and $t_{a}$ (after the policy change). By only considering employed individuals, the analysis removes potential adverse effects of unemployment on health which may (downwardly) bias the results.

Using the natural experiment created by the 2017 NMW change as an example, individuals earning below the April 2017 NMW (£7.50) before April 2017 and earning equal to or above that rate after April 2017 are in the treatment group. The control groups wages in the *before* period must be equal or up to 120% of the April 2017 NMW in the base case analysis. This condition means the control group are not affected by the NMW but are similar in their earnings to the treatment group. There is a trade‐off between the quality of counterfactual and sample size (statistical power). The higher the control groups wages are permitted to be, the further they may diverge from the treatment group in their income and characteristics, although this permits a larger sample. Therefore, a sensitivity analysis using a 140% condition was also implemented in line with Kronenberg et al. (2017). Finally, the control group are permitted to have up to a 20% pay increase between $t_{b}$ and $t_{a}$, as to exclude individuals with large pay increases. If individuals with large pay increases were included, the control group may be a poor counterfactual as both the treated and control individuals receive a pay rise (the former due to the NMW, the latter for other reasons). If higher incomes lead to improved health, the control individual's health will increase following a large pay increase. The Average Treatment Effect for the Treated (ATT) estimate will be downwardly biased, as the ATT estimate also captures increasing health in the control group. Treatment and control group sizes for the base case and presented in Table 1.

**TABLE 1:** Treatment and control group sizes by year, base case

Year	Control group	Treatment group
2016	513	290
2017	614	470
2018	440	363

*Note*: This table gives the number of individuals in the treatment and control groups for the base case analysis. The left‐hand column is the year of the NMW change, and the two right hand columns give the number of individuals in the treatment and control groups when analysing that years NMW change as a natural experiment.

### 2.2 Dependent variables

The dependent variables are the SF‐12 mental and physical component summary scores (MCS and PCS respectively). The SF‐12 is a psychometrically tested generic non‐preference‐based measure of health which contains eight dimensions, such as questions on depression and physical functioning, which are common in most generic health instruments. In the UK general population, SF‐12 scores are transformed to be on a 0–100 scale with a mean of 50 and standard deviation of 10 (Jenkinson, 1999), meaning the score may be used to evaluate differences in health between groups.

The SF‐12 performs well when evaluated against the Brazier and Deverill (1999) checklist of practicality, reliability and validity, meaning it is one of the world's most popular generic health instruments (Brazier et al., 2016). Evidence for practicality is presented in Brazier et al. (2016), whilst Ware et al. (1996) demonstrate reliability over time (test‐retest reliability), across respondents, and across locations. Ware et al. (1996) also demonstrates that the SF‐12 covers all dimensions of interest for general health and is sensitive to changes in general health (content validity). Therefore, the evidence suggests that the SF‐12 demonstrates sufficient practicality, reliability and validity to be suitable for the subsequent analysis. A limitation of the SF‐12, as with any self‐reported measure, is the risk of measurement error. However, as this paper uses panel data, individual's self‐reported health in period *t* will only be compared that same individual's health in period *t* + *1.* Hence the differencing operation should remove this reference bias in the DiD estimate (Lenhart, 2017).

### 2.3 Wages and covariates

Wages are self‐reported and given to the nearest penny meaning individuals subject to the NMW can be identified with a high degree of accuracy. This is an advantage over previous papers, such as Kronenberg et al. (2017), who derive hourly wages from self‐reported monthly income data. This derivation may introduce error as it does not accurately account for the actual hours worked per month. Therefore, individuals may be mis‐allocated to treatment or control groups, leading to spill overs and bias (although the direction of the bias is ambiguous). However, like any self‐reported data, wages reported in Understanding Society may still be subject to measurement error. A later question in Understanding Society asks respondents whether their wage information is an estimate or exact figure. Table 2 demonstrates for the 2016 base case, 77% of respondents in the treatment group reported their exact wage (vs. 79% in the control). Although this may represent a high degree of accuracy, any lack of precision may lead to mis‐allocated treatment and control groups. Covariates are included in the regression specification to increase the precision of ATT estimates and account for differential trends. The empirical estimation deliberately adopts a parsimonious specification of the DiD equation due to concern that including bad controls may bias the estimates of the coefficients on the variables of interest. Only those variables that one can be reasonably confident are themselves exogenous are included; these are age, marital status, and gender.

Tables 2, 3, 4 present the pre‐treatment descriptive statistics for the base case 2016 NMW analysis. The significance stars indicate whether there is a statistical difference in means between the treatment and control groups as estimated by a two‐sampled *t*‐test. If there is no significant difference in means, this suggests the sample is balanced, and, allocation to treatment groups is as‐if random, which indicates the two groups are similar and the control group is a valid counterfactual. Using the 2016 NMW change as an example, Table 2 shows there is a there is a statistically significant difference in the average wage levels before the NMW increase between the groups at the 1% level, which is expected as the allocation of individuals to groups is conditional on their wage being above or below the NMW in a given year (£7.20 in this example). The SF‐12 MCS and PCS scores for each group have a mean of approximately 50 and standard deviation of 10 which suggests the sample is representative because SF‐12 scores are standardized to a mean of 50 and standard deviation of 10 using the UK population (Jenkinson, 1999, Ware et al., 1995). SF‐12 scores between groups are statistically similar suggesting there is no selection bias, although any selection effect would be removed in the differencing operation. The average age of the sample overall is approximately 45 and just over half are married, which broadly mirrors the demography of the UK overall, again giving evidence that the sample is representative the UK population (Office for National Statistics, 2019a, 2019b). Table 2 also shows the control group has a higher mean age which is statistically significant at the 10% level. This is expected given that older individuals generally earn more (Mincer, 1958), and the control group are individuals earning above the minimum wage (rather than below). Over two‐thirds of each group are female which is unsurprising given the gender pay gap. Finally, *Exact wage* shows the proportion of each group whose wage data is an exact amount rather than an estimate. Measurement error may reduce the accuracy of the allocation to treatment and control groups, although there is no statistical difference in the proportion reporting exact wages in the base case, suggesting that measurement error affects both groups equally.

**TABLE 2:** Summary statistics pre‐treatment by group, 2016 NMW base case

	Control group		Treatment group	
	Mean	Std. dev.	Mean	Std. dev.
Wage	7.79***	0.51	6.94***	1.27
SF‐12 MCS	49.31	9.37	49.36	8.90
SF‐12 PCS	50.86	9.44	51.33	9.06
Age	45.72*	10.36	44.26*	11.08
Female	0.69	0.46	0.71	0.45
Married	0.55	0.50	0.49	0.50
Exact wage	0.79	0.41	0.77	0.39
Observations	513		290	

Abbreviations: MCS, mental component summary score; NMW, National Minimum Wage; PCS, physical component summary score.

*significant at 10%, **significant at 5%, ***significant at 1%.

**TABLE 3:** Summary statistics pre‐treatment by group, 2017 NMW base case

	Control group		Treatment group	
	Mean	Std. dev.	Mean	Std. dev.
Wage	8.06***	0.53	7.21***	0.48
SF‐12 MCS	49.13	9.82	48.21	10.24
SF‐12 PCS	50.86	9.19	50.31	9.32
Age	45.40**	10.64	44.10**	10.85
Female	0.69	0.46	0.70	0.46
Married	0.55**	0.50	0.48**	0.50
Exact wage	0.75***	0.43	0.89***	0.34
Observations	614		470	

Abbreviations: MCS, mental component summary score; NMW, National Minimum Wage; PCS, physical component summary score.

*significant at 10%, **significant at 5%, ***significant at 1%.

**TABLE 4:** Summary statistics pre‐treatment by group, 2018 NMW base case

	Control group		Treatment group	
	Mean	Std. dev.	Mean	Std. dev.
Wage	8.45***	0.54	7.53***	0.53
SF‐12 MCS	49.10**	9.24	47.54**	10.32
SF‐12 PCS	51.19	8.75	50.24	9.41
Age	46.15*	10.81	44.67*	11.45
Female	0.66	0.47	0.70	0.46
Married	0.54**	0.50	0.46**	0.50
Exact wage	0.78*	0.41	0.83*	0.38
Observations	440		363	

*Note*: Tables 2, 3, 4 show the mean and standard deviation of several variables split by treatment and control group for the 2016, 2017 and 2018 natural experiments. All values are calculated using data from the *before* period. The significance stars indicate whether there is a statistical difference in means between the treatment and control groups as estimated by a two‐sampled *t*‐test. In the base case analysis, the control groups wages in the before period must be equal or up to 120% of the new NMW rate following the uprating.

Abbreviations: MCS, mental component summary score; NMW, National minimum wage; PCS, physical component summary score.

*significant at 10%, **significant at 5%, ***significant at 1%.

Table 5 shows the top NMW rate in nominal and real terms from 2008 to 2019. Although the NMW has consistently increased in nominal terms since 2008, this does not always translate into real terms increases. It is unlikely that NMW changes will significantly affect health outcomes if they do not increase the purchasing power of an individual's income. Therefore, the analysis focuses on the April 2016, 2017 and 2018 NMW increases, which saw NMW increases above the rate of inflation in that financial year. The October 2013 NMW increase is used as a placebo “test” for common trends, using the assumption that a flat real‐terms NMW change should have no impact on purchasing power and therefore health outcomes.

**TABLE 5:** Nominal and real NMW rates 2008–2019

	Nominal NMW	Real NMW (2018/19 prices)	Real change (%)
01‐Oct‐2008	£5.73	£6.81	‐
01‐Oct‐2009	£5.80	£6.78	−0.42%
01‐Oct‐2010	£5.93	£6.81	0.52%
01‐Oct‐2011	£6.08	£6.88	0.99%
01‐Oct‐2012	£6.19	£6.86	−0.25%
01‐Oct‐2013	£6.31	£6.86	0.01%
01‐Oct‐2014	£6.50	£6.97	1.59%
01‐Oct‐2015	£6.70	£7.13	2.20%
01‐Apr‐2016	£7.20	£7.48	4.98%
01‐Apr‐2017	£7.50	£7.66	2.40%
01‐Apr‐2018	£7.83	£7.83	2.22%
01‐Apr‐2019	£8.21	£8.05	2.86%

*Note*: This table shows the top NMW rate in nominal and real terms from 2008 to 2019. Individuals are eligible for different NMW rates depending on their age. This paper only analyses those affected by the “top rate”, which refers to the NMW rate which the highest age bracket is eligible for. The highest age bracket changes over time. When the NMW changed in October 2008 and 2009 the top rate applied to ages 22+. From October 2010 to October 2015 the top rate applied to ages 21+. From April 2016 onwards the top rate applied to ages 25+. To calculate the real NMW, the nominal NMW adjusted to 2018/19 prices using the Office for National Statistics GDP deflator (2020).

Abbreviation: NMW, National Minimum Wage.

## 3 METHODS

The analysis utilizes a two‐period DiD approach as outlined in Khandker et al. (2010). Here, the difference in observed mean health outcomes between groups is compared in period $t_{b}$, the year before the NMW increase, and period $t_{a}$ *,* the year after. The dates used to allocate respondents data to *before* and *after* periods are outlined in Table 6.

**TABLE 6:** Nominal and real NMW rates 2008–2019

Year	Date of change	Before period	After period
2016	01‐Apr‐2016	01‐Apr‐2015 to 31‐Mar‐2016	01‐Apr‐2016 to 31‐Mar‐2017
2017	01‐Apr‐2017	01‐Apr‐2016 to 31‐Mar‐2017	01‐Apr‐2017 to 31‐Mar‐2018
2018	01‐Apr‐2018	01‐Apr‐2017 to 31‐Mar‐2018	01‐Apr‐2018 to 31‐Mar‐2019

*Note*: This table shows the dates used to allocate respondents data to *before* and *after* periods. For the 2016 change, the policy intervention analysed is the April 2016 NMW uprating. However, in previous years the change had occurred in October (see Table 5), and hence there was also a NMW change in October 2015 (during the *before* period of the 2016 natural experiment). To account for this, all individuals in the treatment or control groups must have a wage higher than the October 2015 NMW (£6.70).

Abbreviation: NMW, National Minimum Wage.

Controlling for confounding factors, the ATT is the change in health that is driven by NMW increases. The regression equation is:

(1) $$Y_{it}=\beta_{0}+\beta_{1}{\text{Post}}_{t}+\beta_{2}{\text{Treated}}_{i}+\beta_{3}\left({\text{Post}}_{t}*{\text{Treated}}_{i}\right)+\beta_{4}^{\prime }X_{it}+\varepsilon_{it}\;t=\left(t_{b},\;t_{a}\right)\;$$

$Y_{it}$ is the dependent variable, set as either the SF‐12 MCS or PCS. ${\mathrm{Post}}_{t}$ indicates the *after* period if it is set to one, or the *before* period if set to zero. ${\mathrm{Treated}}_{i}$ is a time‐invariant binary variable which is set to one if the individual is in the treatment group. The coefficient of interest is $\beta_{3}$, which is the effect size of the interaction between ${\mathrm{Post}}_{t}$ and ${\mathrm{Treated}}_{i}$, or the ATT. $X_{it}$ is a vector of individual level determinants of health (age, marital status, gender), which were informed by the existing literature. A fixed effects specification is used, meaning $\varepsilon_{it}$ is the composite error term, defined as $\varepsilon_{it}=a_{i}+u_{it}$ where $a_{i}$ accounts for time‐invariant unobserved heterogeneity. For example, this might be the unobserved effect of readily available stop smoking services, access to which differs by individual but not between $t_{b}$ and $t_{a}$. This factor may affect health, but data is not available in Understanding Society to include it as a covariate. The fixed effects specification was selected in order to eliminate the unobserved effect $a_{i}$. This is desirable as when using ordinary least squares (OLS) on panel data it must be assumed that covariates and $a_{i}$ are uncorrelated. If this does not hold then pooled OLS estimates will be biased and inconsistent, giving misleading results of the ATT estimate. Importantly, the fixed effects specification means that time invariant covariates, such as gender, will be omitted. However, this is not problematic because the DiD coefficient, $\beta_{3}$, is still analysable as $\left({\mathrm{Post}}_{t}*{\mathrm{Treated}}_{i}\right)$ is time variant. Two assumptions must hold for DiD to produce an unbiased ATT estimate. Firstly, there must be common trends meaning that were the NMW not introduced, the health outcomes of the two groups would follow the same trend. Secondly, there must be no spill overs, meaning NMW increases must not affect the control group. Spill overs would constitute a violation of the *stable unit treatment value assumption* (SUTVA). These assumptions are discussed later.

## 4 RESULTS

### 4.1 Wage as the dependent variable

To estimate the impact of the NMW, the treatment and control groups must be statistically similar except for their exposure to the NMW. This means the treatment group should experience an increase in wages above any wage increases that the control group receive. To establish the wage effect, we run Equation (2):

(2) $${\text{wage}}_{it}=\beta_{0}+\beta_{1}{\text{Post}}_{t}+\beta_{2}{\text{Treated}}_{i}+\beta_{3}\left({\text{Post}}_{t}*{\text{Treated}}_{i}\right)+\beta_{4}^{\prime }X_{it}+\varepsilon_{it}\;t=\left(t_{b},\;t_{a}\right)$$

Table 7 presents the results for impact of the 2016, 2017 and 2018 NMW changes on wages for the base case. The coefficient on Post*Treated is the ATT estimate, and gives the additional change in wages between the before and after periods for the treated group. The model predicts that on average, the 2016 NMW increase led wages in the treatment group to increase by 54p per hour, all other factors held constant, which is statistically significant at the 1% level. Statistically significant increases in wages are also seen in 2017 (34p per hour) and in 2018 (42p per hour). This gives confidence that group allocation has been effective as the treatment group experienced an increase in wages on average (due to the NMW) whilst the control group did not. The health effect of these different wage increases following the NMW uprating is tested in the following section. When analysing the 2017 or 2018 NMW changes, and when using a larger control group as a sensitivity analysis, there is still a statistically significant impact of the NMW on wages (see Appendix A for details of the sensitivity analysis undertaken).

**TABLE 7:** Fixed effects DiD using wage as dependent variable, base case

	2016		2017		2018	
	*β*	(se)	*β*	(se)	*β*	(se)
Post	0.210	(0.150)	0.209***	(0.061)	0.123	(0.079)
Treated	0.000	(.)	0.000	(.)	0.000	(.)
Post*Treated	0.539***	(0.080)	0.342***	(0.030)	0.417***	(0.040)
Age	0.003	(0.143)	0.001	(0.057)	0.069	(0.074)
Married	0.033	(0.199)	−0.118	(0.081)	−0.041	(0.136)
Female	0.000	(.)	0.000	(.)	0.000	(.)
Constant	7.344	(6.449)	7.717***	(2.539)	4.915	(3.387)
Observations	1606		2168		1606	

*Note*: This table reports Average Treatment Effect for the Treated (ATT) estimates and covariates from panel data difference‐in‐differences (DiD) models. The dependent variable is wages. A fixed effects specification is used. The variable of interest is Post*Treated, which is the ATT estimate. In the base case analysis, the control groups wages in the before period must be equal or up to 120% of the new NMW rate following the uprating. We run this regression to ensure that there is a statistically significant impact of NMW upratings in 2016, 2017 and 2018 on the wages of the treatment group. If there is a significant increase in wages following the uprating, then we can later test whether this wage increase translated to improved health.

Abbreviation: DiD, difference‐in‐differences.

*significant at 10%, **significant at 5%, ***significant at 1%.

### 4.2 Health as the dependent variable

Table 8 presents the results from the DiD regressions analysing the impact of the 2016, 2017 and 2018 NMW increases on the SF‐12 MCS and PCS. The table is a summary of the Post*Treated interaction coefficients for the base case, which are the ATT estimates for separate regressions which use the SF‐12 MCS or PCS as the dependent variable. The coefficient on Post*Treated gives the additional change in health between the *before* and *after* periods for the treated group. The model predicts that the 2016 NMW increase led to a 0.274‐point decrease in SF‐12 MCS scores for the treated group, holding other factors constant, which is statistically insignificant at all conventional levels of significance. This suggests there was no significant impact of the 2016 NMW increase on the mental health of individuals in the sample who received the NMW increase. The models also predict insignificant impacts of the 2017 and 2018 NMW increases on mental health. Furthermore, the fixed effects DiD coefficients when using the SF‐12 PCS as the dependent variable, in the 2016, 2017 and 2018 models, are also insignificant at all conventional significance levels. This suggests the NMW changes in all years analyzed had no significant impact on physical health.

**TABLE 8:** Fixed effects DiD estimators by dependent variable and NMW change, base case

Dependent variable	2016		2017		2018	
	*β*	(se)	*β*	(se)	*β*	(se)
SF‐12 MCS	−0.274	(0.675)	0.178	(0.601)	−0.492	(0.661)
SF‐12 PCS	0.043	(0.582)	0.494	(0.502)	0.561	(0.543)
Observations	1606		2168		1606	

*Note*: This table reports Average Treatment Effect for the Treated (ATT) estimates from panel data *c* (DiD) models examining the 2016, 2017 and 2018 NMW upratings as natural experiments. The dependent variables are the SF‐12 MCS or PCS. A fixed effects specification is used. In the base case analysis, the control groups wages in the before period must be equal or up to 120% of the new NMW rate following the uprating.

Abbreviations: DiD, difference‐in‐differences; MCS, mental component summary score; NMW, National Minimum Wage; PCS, physical component summary score.

*significant at 10%, **significant at 5%, ***significant at 1%.

### 4.3 Common trends and robustness checks

**FIGURE 1:**
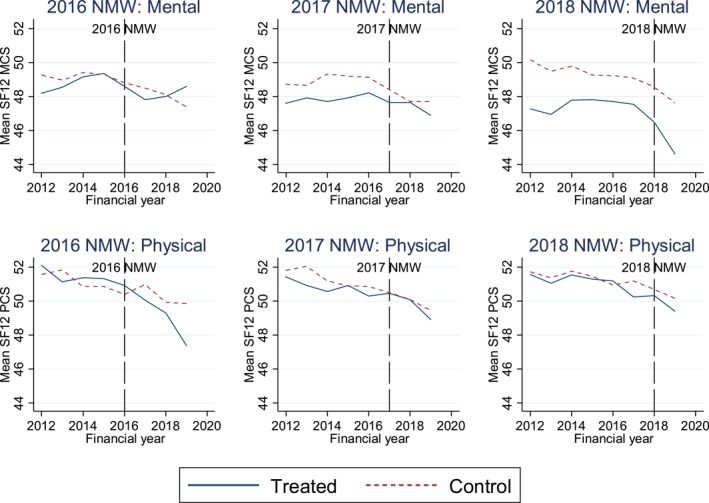
Trends in mean SF‐12 mental component summary scores (MCS) and physical component summary scores (PCS) (base case treatment and control groups). Figure 1 shows the trends in mean SF‐12 MCS and PCS scores for the base case treatment and control groups when examining the 2016, 2017 and 2018 natural experiments. The vertical line indicates when the new NMW rate was introduced for that natural experiment. The treatment and control groups contain the same individuals when examining the MCS and PCS scores

**FIGURE B1:**
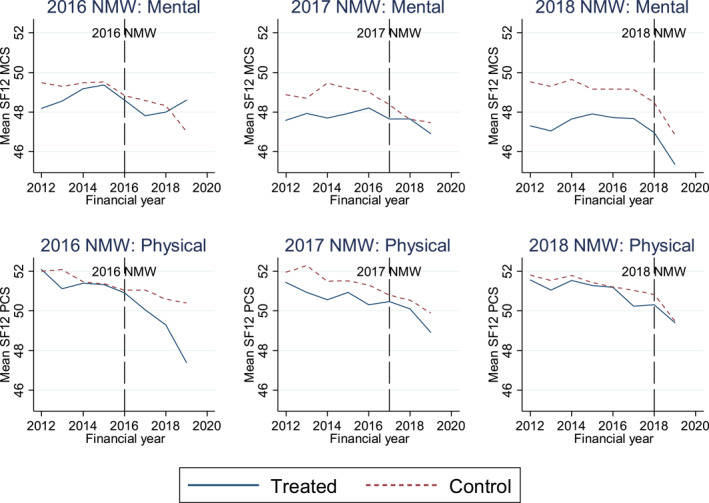
Trends in mean SF‐12 mental component summary scores (MCS) and physical component summary scores (PCS) for treatment and control groups, with larger control group for sensitivity analysis (wages 100%–140% of National Minimum Wage (NMW) in before period). Figure B1 shows the trends in mean SF‐12 MCS and PCS scores for treatment and control groups in 2016, 2017 and 2018, where a larger control group is used for sensitivity analysis (wages 100%–140% of NMW in before period, as opposed to 100%–120% in the base case). The vertical line indicates when the new NMW rate is introduced for that natural experiment. The treatment and control groups contain the same individuals when examining the MCS and PCS scores

There is no formal test for common trends, but the validity of the assumption can be assessed (Gertler et al., 2016). If common trends are not present, the ATT estimate would also capture the effect of differential trends and results will be biased. Notably, differential trends implies $\varepsilon_{it}$ and $\left({\mathrm{Post}}_{t}*{\mathrm{Treated}}_{i}\right)$ are correlated which violates the classic linear regression model assumption of no multicollinearity. If there are common trends, it is not essential that the treatment and control groups have the same average outcomes before the policy, as any selection bias is removed by the differencing operation. Figure 1 shows the trends in mean SF‐12 MCS and PCS scores for the base case treatment and control groups in each year. In Figure 1 the mean SF‐12 scores before the NMW increase (the timing of which is shown by the vertical dashed line) appear to be relatively parallel, albeit with some fluctuation. Appendix B (Figure B1) shows the trends in mean SF‐12 MCS and PCS scores when using larger control groups as a sensitivity analysis. We can have further confidence that common trends hold as the summary statistics in Tables 2, 3, 4 demonstrate that the sample is strongly balanced based on observable factors (albeit with some expected differences in the mean ages of the groups). Therefore, it is likely that any potential differences in trends are driven by random factors. This may be exacerbated by the small sample size in the treatment and control groups, meaning the average SF‐12 scores are potentially more sensitive to outliers in the sample.

To check the robustness of the base case results, two sensitivities are undertaken. First, the criteria used to define the control group is loosened to allow control individuals to have wages up to 140% of the new NMW (see Appendix A for statistical tables). This is to increase the sample size and statistical power. This approach is only undertaken as a sensitivity as it may reduce the comparability of the treatment and control groups as we include individuals further up the income distribution. When analysing either the 2016, 2017 or 2018 NMW changes, the ATT is statistically insignificant at all conventional significance levels. As the results are not sensitive to using the larger control groups, this increases the confidence in the base case finding that there was no significant impact of the 2016, 2017 and 2018 NMW increases on mental or physical health.

Secondly, as a “test” for common trends, a placebo DiD is undertaken. If the placebo DiD were to find a significant ATT estimate in the placebo period, the estimated impact is likely to stem from an underlying difference in trends between the two groups because there is no policy effect for the DiD to register. The 2013 NMW change is used for a placebo DiD, because the October 2013 NMW increase was approximately flat in real terms. There should be no change to the purchasing power of treated individuals, so the NMW increase should have no impact on health. In this analysis, the *before* period is 01‐Oct‐2012 to 30‐Sep‐2013 and the *after* period is 01‐Oct‐2013 to 30‐Sep‐2014. Table 9 presents the placebo DiD coefficients when using the MCS and PCS as the dependent variable respectively.

**TABLE 9:** Fixed effects DiD estimators using placebo 2013 NMW uprating by dependent variable, base case

Dependent variable	2013	
	*β*	(se)
SF‐12 MCS	0.759	(0.696)
SF‐12 PCS	−0.524	(0.596)
Observations	1762	

*Note*: This table reports Average Treatment Effect for the Treated (ATT) estimates from panel data difference‐in‐differences (DiD) models examining the 2013 NMW uprating as a placebo. The dependent variables are the SF‐12 MCS or PCS. A fixed effects specification is used. In the base case analysis, the control groups wages in the before period must be equal or up to 120% of the new NMW rate following the uprating.

Abbreviations: DiD, difference‐in‐differences; MCS, mental component summary score; NMW, National Minimum Wage; PCS, physical component summary score.

*significant at 10%, **significant at 5%, ***significant at 1%.

Table 9 demonstrates there was no statistically significant effect of the NMW change on MCS or PCS scores in the placebo period, which suggests there are common trends in SF‐12 scores. This means standard DiD can be used, rather than a triple‐differences approach.

## 5 DISCUSSION AND CONCLUSION

This paper adds to the existing literature on the health effects of minimum wages by being the first paper to analyse the impact of the NMW on SF‐12 responses, and, by examining the subsequent upratings of the NMW rather than its introduction. The paper exploits the natural experiments provided by UK NMW increases in 2016, 2017 and 2018 to analyse the impact of higher minimum wages on self‐reported mental and physical health. A DiD approach is undertaken using The UK Household Longitudinal Study (Understanding Society), whilst justification is provided to argue that common trends hold.

In the base case and all sensitivity analyses, the ATT estimates for the impact of the 2016, 2017 and 2018 NMW increases on mental health are insignificant. These findings align to those of Kronenberg et al. (2017) who find no significant impact of the NMW on mental health. The paper demonstrates that the NMW significantly increased the wages of the treatment group. However, the evidence suggests that these wage increases did not translate into improved mental health through, for example, reduced financial stress as suggested by Lenhart (2017). One hypothesis is that larger wage increases would lead to more significant health effects. However, the larger real‐terms NMW increase in 2016 was still found to have an insignificant impact on SF‐12 scores. The analysis also found insignificant effects of the 2016, 2017 and 2018 NMW increases on physical health. These findings are broadly in line with the existing literature (Reeves et al., 2017). This suggests the potential mechanisms behind the NMW impact on physical health, such as those described in empirical papers such as Lenhart (2017), are not as convincing as one may have originally thought. Although increased wages may increase healthy behaviors such as fruit and vegetable consumption, the same can be said for risky behaviors such as smoking. Furthermore, it may be that physical health investments manifest themselves over the long term, which would not be registered in these results.

A limitation of the analysis is that the common trends assumption may not hold, particularly in the pre‐treatment trends of mental and physical health for the 2016 NMW change (Figure 1). Although the treatment and control group samples appear to be balanced, and a placebo DiD is undertaken, these are not definitive tests for common trends, and therefore the corresponding DiD estimates should be interpreted with caution. A further limitation is that the regressions use small samples, which is not uncommon in the literature (Reeves et al., 2017). The issue stems from one of the papers strengths, in that strict definitions are used to allocate individuals to treatment and control groups, to minimize the risk of selection bias. Sensitivity analyses using larger control groups found no change to the significance of the results with a larger sample size. A final limitation may be that spill overs of the treatment effect onto the control group may lead to a violation of the SUTVA, as some households may contain both a treatment and a control individual. The effect of this may be to bias the results towards zero. Potential mitigations may be to remove these households or restrict the sample to single‐earner households.

There are several potential avenues for future research. Firstly, an exploration of within‐household spill over effects may prove to be an important factor affecting ATT estimates. Secondly, analysis of the long‐term health effects of the NMW may provide interesting results that are not captured in this paper or in the literature. Thirdly, there may be heterogenous treatment effects across sub‐populations, which may be of future interest. Finally, the introduction of the National Living Wage in April 2016 also entailed changes in the eligibility age for the top wage rate. This provides an exogenous age cut‐off for which regression discontinuity design could be applied to examine the impact of differing NMW rates on young people (Dickens et al., 2014). There is also the possibility that the balance of treatment and control groups may be further enhanced through pre‐processing data through matching, which may be considered as part of further research.

The wider societal impacts of the NMW are still not fully understood. Given the findings of this analysis, and the mixed results found in the literature, at present the policy implication of this work is that health effects of the NMW should not be included in future cost‐benefit analyses examining the NMW.

## CONFLICT OF INTEREST

There are no conflict of interest.

## Data Availability

The data that support the findings of this study are openly available from Understanding Society. Ref: University of Essex, Institute for Social and Economic Research, NatCen Social Research, Kantar Public. (2019). Understanding Society: Waves 1–9, 2009–2018 and Harmonized BHPS: Waves 1–18, 1991–2009. [Data set]. 12th Edition. UK Data Service. SN: 6614, http://doi.org/10.5255/UKDA‐SN‐6614‐13.

## APPENDIX A STATISTICAL TABLES

This appendix presents statistical tables (Tables A1, A2, A3, A4, A5, A6, A7, A8, A9, A10, A11, A12) which were not presented in the main text of the paper.

**TABLE A1:** Treatment and control group sizes by year, using larger control group for sensitivity analysis (wages 100%–140% of NMW in before period)

Year	Control group	Treatment group
2016	756	290
2017	875	470
2018	656	363

*Note*: This table gives the number of individuals in the treatment and control groups for the base case analysis. The left‐hand column is the year of the NMW change, and the two right hand columns give the number of individuals in the treatment and control groups when analysing that years NMW change as a natural experiment.

Abbreviation: NMW, National Minimum Wage.

**TABLE A2:** Summary statistics pre‐treatment by group, 2016 NMW using larger control group for sensitivity analysis (wages 100%–140% of NMW in before period)

	Control group		Treatment group	
	Mean	Std. dev.	Mean	Std. dev.
Wage	8.30***	0.90	6.94***	1.27
SF‐12 MCS	49.53	9.28	49.36	8.90
SF‐12 PCS	51.38	8.91	51.33	9.06
Age	45.93**	10.10	44.26**	11.08
Female	0.64**	0.48	0.71**	0.45
Married	0.57**	0.50	0.49**	0.50
Exact wage	0.77	0.42	0.77	0.39
Observations	756		290	

*Note*: The table shows the mean and standard deviation of several variables split by treatment and control group for the 2016 natural experiment. All values are calculated using data from the *before* period. The significance stars indicate whether there is a statistical difference in means between the treatment and control groups as estimated by a two‐sampled *t*‐test. In this sensitivity analysis, the control groups wages in the before period must be equal or up to 140% of the new NMW rate following the uprating (rather than 120% in the base case analysis), which increases the size of the control group.

Abbreviations: MCS, mental component summary score; NMW, National Minimum Wage; PCS, physical component summary score.

*significant at 10%, **significant at 5%, ***significant at 1%.

**TABLE A3:** Summary statistics pre‐treatment by group, 2017 NMW using larger control group for sensitivity analysis (wages 100%–140% of NMW in before period)

	Control group		Treatment group	
	Mean	Std. dev.	Mean	Std. dev.
Wage	8.56***	0.92	7.21***	0.48
SF‐12 MCS	49.02	9.97	48.21	10.24
SF‐12 PCS	51.31*	8.96	50.31*	9.32
Age	45.52**	10.31	44.10**	10.85
Female	0.66*	0.47	0.70*	0.46
Married	0.57***	0.49	0.48***	0.50
Exact wage	0.75***	0.44	0.89***	0.34
Observations	875		470	

*Note*: The table shows the mean and standard deviation of several variables split by treatment and control group for the 2017 natural experiment. All values are calculated using data from the *before* period. The significance stars indicate whether there is a statistical difference in means between the treatment and control groups as estimated by a two‐sampled *t*‐test. In this sensitivity analysis, the control groups wages in the before period must be equal or up to 140% of new NMW rate following the uprating (rather than 120% in the base case analysis), which increases the size of the control group.

Abbreviations: MCS, mental component summary score; NMW, National Minimum Wage; PCS, physical component summary score.

*significant at 10%, **significant at 5%, ***significant at 1%.

**TABLE A4:** Summary statistics pre‐treatment by group, 2018 NMW using larger control group for sensitivity analysis (wages 100%–140% of NMW in before period)

	Control group		Treatment group	
	Mean	Std. dev.	Mean	Std. dev.
Wage	8.96***	0.91	7.53***	0.91
SF‐12 MCS	49.13**	9.56	47.54**	9.56
SF‐12 PCS	51.05	8.79	50.24	8.79
Age	46.30**	10.52	44.67**	10.52
Female	0.63**	0.48	0.70**	0.48
Married	0.56***	0.50	0.46***	0.50
Exact wage	0.76**	0.42	0.83**	0.42
Observations	656		363	

*Note*: The table shows the mean and standard deviation of several variables split by treatment and control group for the 2018 natural experiment. All values are calculated using data from the *before* period. The significance stars indicate whether there is a statistical difference in means between the treatment and control groups as estimated by a two‐sampled *t*‐test. In this sensitivity analysis, the control groups wages in the before period must be equal or up to 140% of new NMW rate following the uprating (rather than 120% in the base case analysis), which increases the size of the control group.

Abbreviations: MCS, mental component summary score; NMW, National Minimum Wage; PCS, physical component summary score.

*significant at 10%, **significant at 5%, ***significant at 1%.

**TABLE A5:** Summary statistics pre‐treatment by group, 2013 placebo base case

	Control group		Treatment group	
	Mean	Std. dev.	Mean	Std. dev.
Wage	8.94	24.51	6.17	28.91
SF‐12 MCS	49.77	9.30	48.84	9.30
SF‐12 PCS	51.40	8.36	51.60	8.19
Age	44.72***	10.25	42.55***	10.61
Female	0.72	0.45	0.69	0.46
Married	0.57*	0.49	0.50*	0.50
Exact wage	0.83	0.39	0.82	0.39
Observations	640		241	

*Note*: The table shows the mean and standard deviation of several variables split by treatment and control group for the 2013 NMW uprating. All values are calculated using data from the *before* period. The significance stars indicate whether there is a statistical difference in means between the treatment and control groups as estimated by a two‐sampled *t*‐test. In the base case analysis, the control groups wages in the before period must be equal or up to 120% of new NMW rate following the uprating.

Abbreviations: MCS, mental component summary score; PCS, physical component summary score.

*significant at 10%, **significant at 5%, ***significant at 1%.

**TABLE A6:** Fixed effects DiD using wage as dependent variable with larger control group for sensitivity analysis (wages 100%–140% of NMW in before period)

	2016		2017		2018	
	*β*	(se)	*β*	(se)	*β*	(se)
Post	0.181	(0.123)	0.176***	(0.058)	0.148**	(0.075)
Treated	0.000	(.)	0.000	(.)	0.000	(.)
Post*Treated	0.582***	(0.070)	0.373***	(0.030)	0.441***	(0.039)
Age	−0.012	(0.118)	0.002	(0.053)	0.020	(0.071)
Married	0.086	(0.173)	−0.083	(0.078)	−0.050	(0.112)
Female	0.000	(.)	0.000	(.)	0.000	(.)
Constant	8.418	(5.369)	8.022***	(2.389)	7.545**	(3.253)
Observations	2092		2690		2038	

*Note*: This table reports Average Treatment Effect for the Treated (ATT) estimates and covariates from panel data difference‐in‐differences (DiD) models. The dependent variable is wages. A fixed effects specification is used. The variable of interest is Post*Treated, which is the ATT estimate. In this sensitivity analysis, the control groups wages in the before period must be equal or up to 140% of new NMW rate following the uprating (rather than 120% in the base case analysis), which increases the size of the control group. We run this regression to ensure that there is a statistically significant impact of NMW upratings in 2016, 2017 and 2018 on the wages of the treatment group. If there is a significant increase in wages following the uprating, then we can later test whether this wage increase translated to improved health.

Abbreviations: DiD, difference‐in‐differences; NMW, National Minimum Wage.

*significant at 10%, **significant at 5%, ***significant at 1%.

**TABLE A7:** Fixed effects DiD regressions using SF‐12 MCS as the dependent variable, base case

	2016		2017		2018	
	*β*	(se)	*β*	(se)	*β*	(se)
Post	0.706	(1.270)	−0.240	(1.218)	−0.180	(1.312)
Treated	0.000	(.)	0.000	(.)	0.000	(.)
Post*Treated	−0.274	(0.675)	0.178	(0.601)	−0.492	(0.661)
Age	−1.244	(1.206)	−0.488	(1.129)	−0.354	(1.235)
Married	3.181*	(1.682)	0.341	(1.617)	−1.624	(2.263)
Female	0.000	(.)	0.000	(.)	0.000	(.)
Constant	103.892*	(54.501)	70.461	(50.676)	65.302	(56.212)
Observations	1606		2168		1606	

*Note*: This table reports Average Treatment Effect for the Treated (ATT) estimates and covariates from panel data difference‐in‐differences (DiD) models examining the 2016, 2017 and 2018 NMW upratings as natural experiments. The dependent variable is the SF‐12 MCS. A fixed effects specification is used. The variable of interest is Post*Treated, which is the ATT estimate. In the base case analysis, the control groups wages in the before period must be equal or up to 120% of new NMW rate following the uprating.

Abbreviations: DiD, difference‐in‐differences; MCS, mental component summary score.

*significant at 10%, **significant at 5%, ***significant at 1%.

**TABLE A8:** Fixed effects DiD regressions using SF‐12 PCS as the dependent variable, base case

	2016		2017		2018	
	*β*	(se)	*β*	(se)	*β*	(se)
Post	−1.026	(1.096)	−0.087	(1.017)	−0.224	(1.079)
Treated	0.000	(.)	0.000	(.)	0.000	(.)
Post*Treated	0.043	(0.582)	0.494	(0.502)	0.561	(0.543)
Age	0.576	(1.041)	−0.225	(0.942)	−0.273	(1.015)
Married	−0.139	(1.451)	−2.414*	(1.350)	0.939	(1.860)
Female	0.000	(.)	0.000	(.)	0.000	(.)
Constant	25.051	(47.037)	61.983	(42.287)	62.680	(46.211)
Observations	1606		2168		1606	

*Note*: This table reports Average Treatment Effect for the Treated (ATT) estimates and covariates from panel data difference‐in‐differences (DiD) models examining the 2016, 2017 and 2018 NMW upratings as natural experiments. The dependent variable is the SF‐12 PCS. A fixed effects specification is used. The variable of interest is Post*Treated, which is the ATT estimate. In the base case analysis, the control groups wages in the before period must be equal or up to 120% of new NMW rate following the uprating.

Abbreviations: DiD, difference‐in‐differences; PCS, physical component summary score.

*significant at 10%, **significant at 5%, ***significant at 1%.

**TABLE A9:** Fixed effects DiD regressions using SF‐12 MCS as the dependent variable, with larger control group for sensitivity analysis (wages 100%–140% of NMW in before period)

	2016		2017		2018	
	*β*	(se)	*β*	(se)	*β*	(se)
Post	0.453	(1.105)	−1.047	(1.081)	−0.365	(1.174)
Treated	0.000	(.)	0.000	(.)	0.000	(.)
Post*Treated	−0.084	(0.627)	0.112	(0.569)	−0.199	(0.608)
Age	−1.173	(1.063)	0.374	(0.995)	−0.465	(1.119)
Married	2.544	(1.559)	0.166	(1.471)	−1.067	(1.760)
Female	0.000	(.)	0.000	(.)	0.000	(.)
Constant	101.439**	(48.320)	31.796	(44.848)	70.373	(51.202)
Observations	2092		2690		2038	

*Note*: This table reports Average Treatment Effect for the Treated (ATT) estimates and covariates from panel data difference‐in‐differences (DiD) models examining the 2016, 2017 and 2018 NMW upratings as natural experiments. The dependent variable is the SF‐12 MCS. A fixed effects specification is used. The variable of interest is Post*Treated, which is the ATT estimate. In this sensitivity analysis, the control groups wages in the before period must be equal or up to 140% of new NMW rate following the uprating (rather than 120% in the base case analysis), which increases the size of the control group.

Abbreviations: DiD, difference‐in‐differences; MCS, mental component summary score; NMW, National Minimum Wage.

*significant at 10%, **significant at 5%, ***significant at 1%.

**TABLE A10:** Fixed effects DiD regressions using SF‐12 PCS as the dependent variable, with larger control group for sensitivity analysis (wages 100%–140% of NMW in before period)

	2016		2017		2018	
	*β*	(se)	*β*	(se)	*β*	(se)
Post	−0.772	(0.928)	−0.622	(0.879)	−0.749	(0.971)
Treated	0.000	(.)	0.000	(.)	0.000	(.)
Post*Treated	−0.088	(0.527)	0.655	(0.463)	0.283	(0.503)
Age	0.456	(0.893)	0.142	(0.810)	0.517	(0.926)
Married	−0.386	(1.310)	−2.058*	(1.197)	1.154	(1.456)
Female	0.000	(.)	0.000	(.)	0.000	(.)
Constant	30.836	(40.611)	45.663	(36.490)	26.505	(42.362)
Observations	2092		2690		2038	

*Note*: This table reports Average Treatment Effect for the Treated (ATT) estimates and covariates from panel data difference‐in‐differences (DiD) models examining the 2016, 2017 and 2018 NMW upratings as natural experiments. The dependent variable is the SF‐12 PCS. A fixed effects specification is used. The variable of interest is Post*Treated, which is the ATT estimate. In this sensitivity analysis, the control groups wages in the before period must be equal or up to 140% of new NMW rate following the uprating (rather than 120% in the base case analysis), which increases the size of the control group.

Abbreviations: DiD, difference‐in‐differences; NMW, National Minimum Wage; PCS, physical component summary score.

*significant at 10%, **significant at 5%, ***significant at 1%.

**TABLE A11:** Fixed effects DiD regressions with SF‐12 MCS as the dependent variable, using placebo 2013 NMW uprating, base case

	2013	
	*β*	(se)
Post	2.137*	(1.202)
Treated	0.000	(.)
Post*Treated	0.760	(0.696)
Age	−2.170*	(1.136)
Married	−1.028	(1.974)
Female	0.000	(.)
Constant	145.834***	(50.185)
Observations	1762	

*Note*: This table reports the Average Treatment Effect for the Treated (ATT) estimate and covariates from a panel data difference‐in‐differences (DiD) model examining the 2013 NMW uprating as a placebo. The dependent variable is the SF‐12 MCS. A fixed effects specification is used. In the base case analysis, the control groups wages in the before period must be equal or up to 120% of new NMW rate following the uprating.

Abbreviations: DiD, difference‐in‐differences; MCS, mental component summary score; NMW, National Minimum Wage.

*significant at 10%, **significant at 5%, ***significant at 1%.

**TABLE A12:** Fixed effects DiD regressions with SF‐12 PCS as the dependent variable, using placebo 2013 NMW uprating, base case

	2013	
	*β*	(se)
Post	−1.752*	(1.030)
Treated	0.000	(.)
Post*Treated	−0.524	(0.596)
Age	1.941**	(0.972)
Married	−2.169	(1.690)
Female	0.000	(.)
Constant	−33.012	(42.977)
Observations	1762	

*Note*: This table reports the Average Treatment Effect for the Treated (ATT) estimate and covariates from a panel data difference‐in‐differences (DiD) model examining the 2013 NMW uprating as a placebo. The dependent variable is the SF‐12 PCS. A fixed effects specification is used. In the base case analysis, the control groups wages in the before period must be equal or up to 120% of the NMW following the uprating.

Abbreviations: DiD, difference‐in‐differences; NMW, National Minimum Wage; PCS, Physical component summary score.

*significant at 10%, **significant at 5%, ***significant at 1%.

Wage regressions use the same definitions for treatment and control groups as the health regressions. For example, the 2016 base case wage regression uses the same treatment and control groups as the 2016 base case MCS and PCS regressions. Hence the summary statistics are the same for the wage and health regressions. We also include a sensitivity analysis where control individuals are permitted to have pre‐treatment wages between 100% and 140% of the new NMW (as opposed to the base case which is set to 120%).

## APPENDIX B COMMON TRENDS WHEN USING LARGER CONTROL GROUP FOR SENSITIVITY ANALYSIS (WAGES 100%–140% OF NMW IN BEFORE PERIOD)

In this sensitivity analysis, the control group definition is loosened to allow control individuals to have wages in period *t* less than or equal to 140% of the minimum wage in period *t* + *1* (rather than 120% in the base case)*,* in line with Kronenberg et al. (2017).
